# Tyrosine phosphorylation of RNA polymerase II CTD is associated with antisense promoter transcription and active enhancers in mammalian cells

**DOI:** 10.7554/eLife.02105

**Published:** 2014-05-09

**Authors:** Nicolas Descostes, Martin Heidemann, Lionel Spinelli, Roland Schüller, Muhammad Ahmad Maqbool, Romain Fenouil, Frederic Koch, Charlène Innocenti, Marta Gut, Ivo Gut, Dirk Eick, Jean-Christophe Andrau

**Affiliations:** 1 Centre d'Immunologie de Marseille-Luminy, Université Aix-Marseille, Marseille, France; 2 Centre National de la Recherche Scientifique (CNRS) UMR6102, Marseille, France; 3 Department of Molecular Epigenetics, Helmholtz Center Munich, Center of Integrated Protein Science Munich, Munich, Germany; 4 Centre Nacional D'Anàlisi Genòmica, Barcelona, Spain; 5 Inserm U631, Marseille, France; 6 Institut de Génétique Moléculaire de Montpellier (IGMM), CNRS-UMR5535, Montpellier, France; Howard Hughes Medical Institute, New York University School of Medicine, United States

**Keywords:** RNA polymerase II, carboxyl terminal domain, antisense transcription, enhancers, initiation, transcription, human

## Abstract

In mammals, the carboxy-terminal domain (CTD) of RNA polymerase (Pol) II consists of 52 conserved heptapeptide repeats containing the consensus sequence Tyr1-Ser2-Pro3-Thr4-Ser5-Pro6-Ser7. Post-translational modifications of the CTD coordinate the transcription cycle and various steps of mRNA maturation. Here we describe Tyr1 phosphorylation (Tyr1P) as a hallmark of promoter (5′ associated) Pol II in mammalian cells, in contrast to what was described in yeast. Tyr1P is predominantly found in antisense orientation at promoters but is also specifically enriched at active enhancers. Mutation of Tyr1 to phenylalanine (Y1F) prevents the formation of the hyper-phosphorylated Pol IIO form, induces degradation of Pol II to the truncated Pol IIB form, and results in a lethal phenotype. Our results suggest that Tyr1P has evolved specialized and essential functions in higher eukaryotes associated with antisense promoter and enhancer transcription, and Pol II stability.

**DOI:** http://dx.doi.org/10.7554/eLife.02105.001

## eLife digest

When a gene is expressed, the DNA is first transcribed to produce an intermediate molecule called a messenger RNA (mRNA), which is then translated to produce a protein. RNA Polymerase II is an enzyme that makes mRNA molecules in organisms as diverse as plants, animals, and yeast.

RNA Polymerase II is a complex made of a number of proteins. The largest protein in this complex includes a ‘carboxy-terminal domain’ that has multiple repeats of seven amino acids one after the other. The first amino acid in each repeat, a tyrosine, is referred to as tyrosine-1. Adding various chemical tags to the amino acids in these repeats co-ordinates the steps involved in the transcription of genes. In yeast, for example, adding a phosphate group to tyrosine-1 seems to help the polymerase to proceed to make long mRNA molecules. However, it is not known what these chemical tags do in humans or other animals.

Now Descostes, Heidemann et al. (and independently Hsin et al.) have shown that the same phosphate groups on tyrosine-1 perform functions in vertebrates (animals with backbones) that are different to those performed in yeast. These functions include protecting the carboxy-terminal domain from being broken down inside cells, and transcribing the DNA that is upstream of genes.

Descostes, Heidemann et al. found that in human cells, RNA Polymerase II with phosphate groups on tyrosine-1 tends to bind to the beginning of genes. However, rather than moving along each gene and transcribing it, the polymerase then moves in the opposite (or ‘antisense’) direction to transcribe the DNA that is upstream of the gene. In most cases, however, the transcription of these ‘upstream antisense RNAs’ does not make a functional RNA molecule and transcription is paused. Furthermore, Descostes, Heidemann et al. found that when RNA Polymerase II that is not tagged with these phosphate groups is degraded in human cells, these cells rapidly die.

Descostes, Heidemann et al. also found that RNA Polymerase II with phosphate tags on tyrosine-1 also binds to, and transcribes, sections of DNA called ‘enhancers’, which are outside of the genes but that help to activate nearby genes. Importantly, these transcribed enhancers are those that work to define the type of cell and tissue—for example a white blood cell—that any given cell will become.

Future studies should help to answer remaining questions such as: how do these chemical tags affect the transcription of genes that are specific to certain tissue types? And do these tags on RNA Polymerase II help to direct cells to become specific cell types?

**DOI:** http://dx.doi.org/10.7554/eLife.02105.002

## Introduction

The activity of RNA Polymerase (Pol) II is responsible for transcription of mRNAs and many noncoding RNAs. Essential for Pol II function is the carboxy-terminal domain (CTD) of its largest subunit Rpb1 that consists of a highly conserved YSPTSPS heptad repetition (Buratowski, 2009; Heidemann et al., 2012). Post-translational modifications (PTMs) of the CTD coordinate both transcription cycle transitions and loading of RNA processing complexes. In the recent years, novel PTMs were described in addition to the well-known Ser5P and Ser2P associated with early transcription and elongation, respectively. These include Ser7P, involved in snRNA gene transcription (Chapman et al., 2007; Egloff et al., 2007), Thr4P associated to transcription elongation in mammals (Hintermair et al., 2012) and to histone gene transcription in chicken (Hsin et al., 2011), and Tyr1P that in yeast is found at gene body locations, consistent with a role in transcription elongation (Mayer et al., 2012). This latter modification remains however so far uncharacterized in mammalian cells and we aimed at deciphering its function in human cells using biochemical and genome-wide approaches.

## Results and discussion

**Figure 1.**
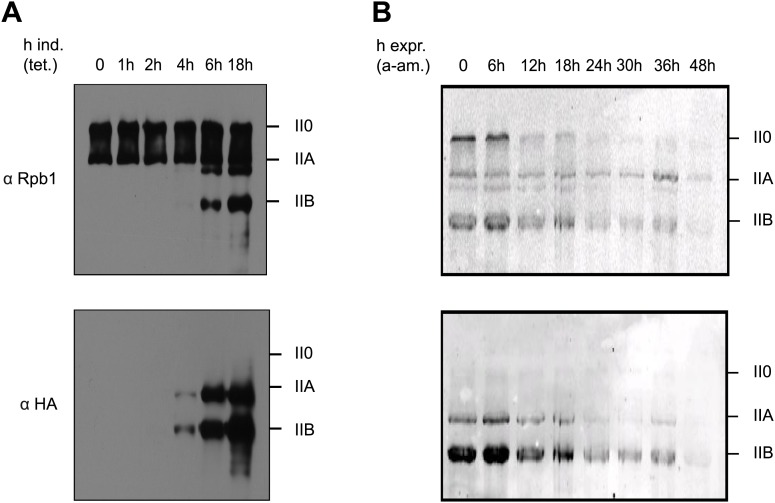
Y1F mutations of the CTD heptads yield a truncated Pol IIB Rpb1. (**A**) Rpb1-Y1F mutant was expressed after removal of tetracycline and in the presence of endogenous Rpb1. Probing with Rpb1 Ab reveals both endogenous and recombinant Rpb1 whereas HA reveals only recombinant Y1F mutant. (**B**) Protein expression of the Y1F mutant after shut-down of endogenous Rpb1 following treatment with α-amanitin. **DOI:** http://dx.doi.org/10.7554/eLife.02105.003

To analyze expression and pattern of Tyr1P modified Pol II, we took advantage of our previously developed Tyr1P specific antibodies (3D12) (Mayer et al., 2012). We investigated various mouse and human cells and could detect Tyr1P in western blots for all examined lines, in most cases associated with the hyper-phosphorylated IIO form of Pol II (Figure 1—figure supplement 1). To address the function of Tyr1P, we next generated Raji cell lines expressing Pol II resistant to α-amanitin (Chapman et al., 2004) and carrying either wild-type (WT) or a mutant Rpb1 gene with substitution of tyrosine to phenylalanine (Y1F) in CTD repeats 4 to 51 (Figure 1—figure supplement 2). After expression of the mutant, we observed that Y1F yielded a truncated Rpb1 (Pol IIB, Figure 1A) and was unable to form the hyper-phosphorylated IIO Pol II. After disruption of the activity of endogenous Pol II by α-amanitin (Figure 1B) and soon after disappearance of WT Rpb1, cells became rapidly inviable. This phenotype reveals an essential function of the Y1 residue that appears more drastic than T4A or S7A mutations, but comparable with that of S5A (Chapman et al., 2007; Hintermair et al., 2012). We conclude that Tyr1P very likely contributes to stabilization of CTD and may occur early within the transcription cycle.

**Figure 1—figure supplement 1.**
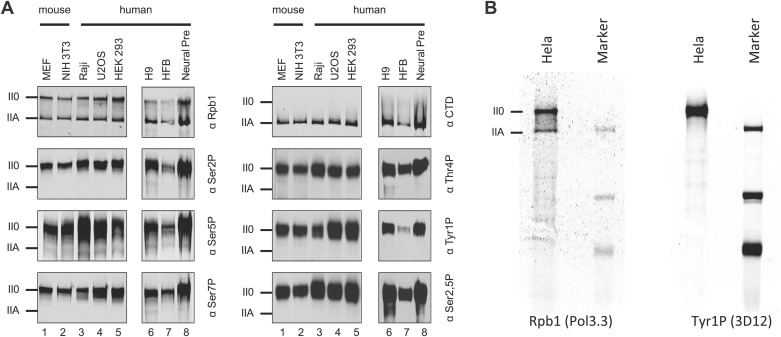
Tyr1P is expressed in various human and mouse cell lines. (**A**) Western blot analyses of antibody recognition in mouse and human cell lines of Rpb1, CTD (8WG16), and CTD isoforms including Tyr1P (3D12). MEF, mouse embryo fibroblasts; Raji, Burkitt-Lymphoma; U2OS, osteosarcoma cell line; HEK293; human embryonic kidney cells; H9, human embryonic stem cells; HFB, human skin fibroblasts; Neural Pre, human neural precursor cells. (**B**) Western blot, as in (**A**) showing the specificity of 3D12 in Hela whole cell extracts over a wider range of proteins. **DOI:** http://dx.doi.org/10.7554/eLife.02105.004

**Figure 1—figure supplement 2.**
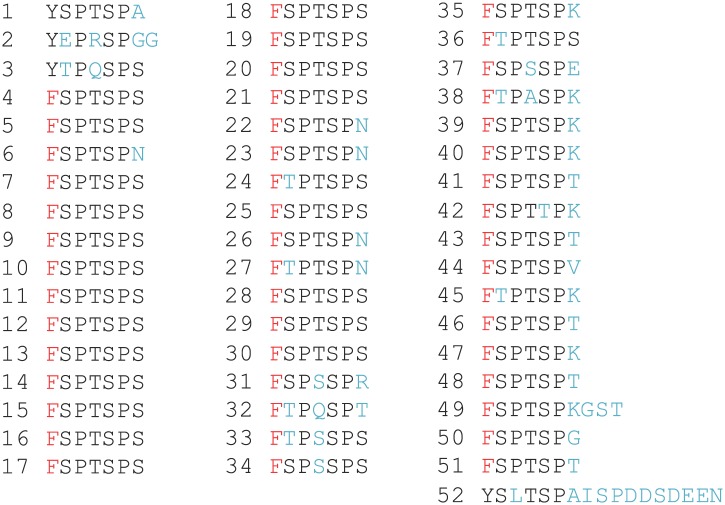
Sequence of the CTD heptads for the Tyr1 to Phe mutant (Y1F). Amino-acid composition of the C-terminal domain of the Y1F mutant (as described in the ‘Materials and methods–Construction of the CTD Y1F mutant’) used for phenotypic and western blot analyses (Figure 1). **DOI:** http://dx.doi.org/10.7554/eLife.02105.005

**Figure 2.**
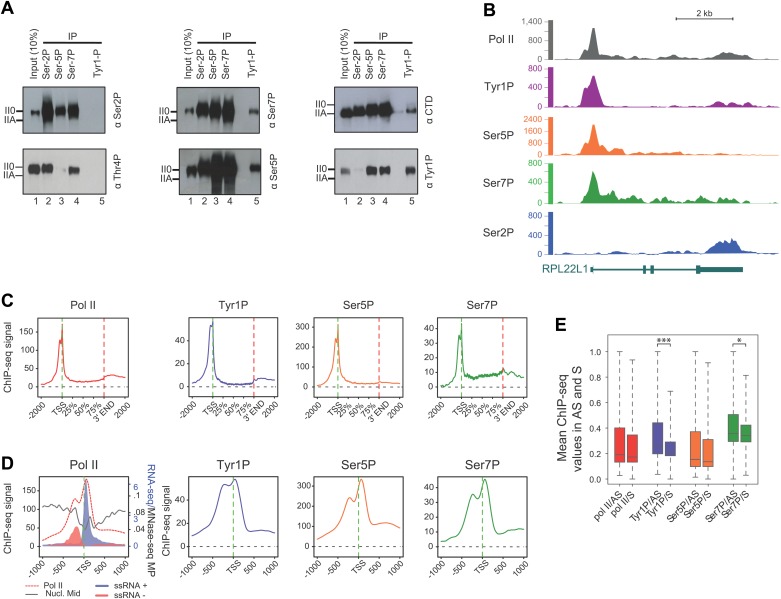
CTD Tyrosine 1 is phosphorylated mainly at TSS and is dominant in antisense transcription. (**A**) Co-immunoprecipitation with specific CTD isoforms in Raji B-cells reveals Tyr1P (3D12) association with Ser5P and Ser7P but not with Ser2P and Thr4P. (**B**) ChIP-seq example illustrating Tyr1P (3D12) association around the promoter of RPL22L1 gene. (**C**) Composite average profiling of ChIP-seq data at coding genes locations for Pol II (1433 genes), Tyr1P (3D12, 2462 genes), Ser5P (1464 genes), and Ser7P (2186 genes) in Raji B-cells and based on selections described in Figure 2—figure supplement 1B. Less stringent selections with more genes gave equivalent profiling (Figure 2—figure supplement 4A). (**D**) Profiling of Pol II, Tyr1P (3D12), Ser5P, Ser7P, nucleosomes midpoint and short strand specific RNAs (ssRNAs) around TSS locations with same selections described in (**C**). (**E**) Boxplots on 3201 genes without outliers showing mean levels of Pol II (2986 genes), Tyr1P (2964 genes), Ser5P (2909 genes), and Ser7P (2948 genes) ChIP-seq signal on regions representing each transcription orientation. The p-values (parametric two sided paired *t* test) of the difference of AS vs S signal are Pol II = 0.5, Tyr1p=3.4 × 10^−15^, Ser5p=0.6, Ser7p=3.5 × 10^−2^. **DOI:** http://dx.doi.org/10.7554/eLife.02105.006

To gain further insight into the involvement of Tyr1P in the transcription cycle, we performed co-immunoprecipitation experiments in human cells using antibodies directed against various CTD modifications reflecting 5′ transcriptionally engaged (Ser5P, Ser7P) or elongating forms (Ser2P, Thr4P) of Pol II (Chapman et al., 2007; Hintermair et al., 2012; Mayer et al., 2012). Our experiments indicated clearly that Tyr1P co-immunoprecipitated with Ser5P and Ser7P but not Ser2P or Thr4P (Figure 2A). Consistently, signals for Tyr1P were observed in Ser5P and Ser7P but not in Ser2P co-immunoprecipitations. Thus, overall, this data points out an association of Tyr1P with early transcribing isoforms of human Pol II.

**Figure 2—figure supplement 1.**
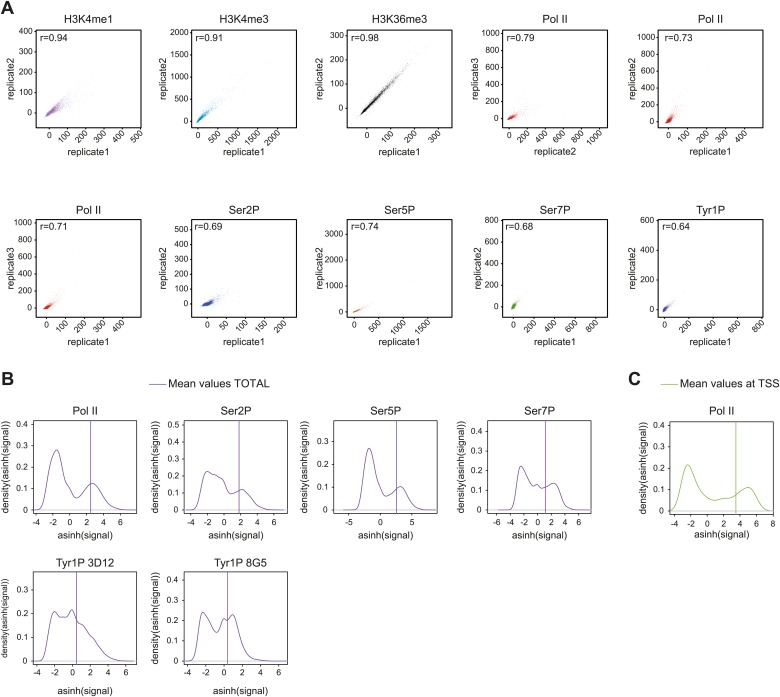
Reproducibility of ChIP-seq experiments and selection of relevant signals used for analyses. (**A**) Correlation plots of biological replicates (for all but H3K36me3 i.e., a technical replicate) of ChIP-seq experiments used in this study at gene locations (‘Materials and methods–Correlation of biological replicates and cross-correlation’). Spearman correlation coefficient is indicated on the top left of the plots. (**B**) Distribution and threshold of background-subtracted signal used for profiling of significantly bound gene (Total, i.e., whole genic regions) in Figure 2, Figure 2—figure supplement 5A, and Figure 2—figure supplement 7C. The mean values used for distribution were computed on [TSS-1000 bp:TES+2000 bp] (TSS: transcription start site; TES: transcription end site). Note that the thresholds were set to the mean of the second Gaussian of the distribution (‘Materials and methods–Gene selection and average binding profiles’). Numbers of genes selected for Pol II, Ser2P, Ser5P, Ser7P, Tyr1P 3D12, and Tyr1P 8G5 are 1521, 1536, 1543, 2382, 2652, and 2608, respectively. (**C**) Distribution and threshold of Pol II significantly bound promoters (TSS) as in (**B**). The selection is used in Figure 3, Figure 3—figure supplement 1, and Figure 3—figure supplement 2. 2044 genes were selected based on their mean values on TSS −/+ 500 bp. **DOI:** http://dx.doi.org/10.7554/eLife.02105.007

**Figure 2—figure supplement 2.**
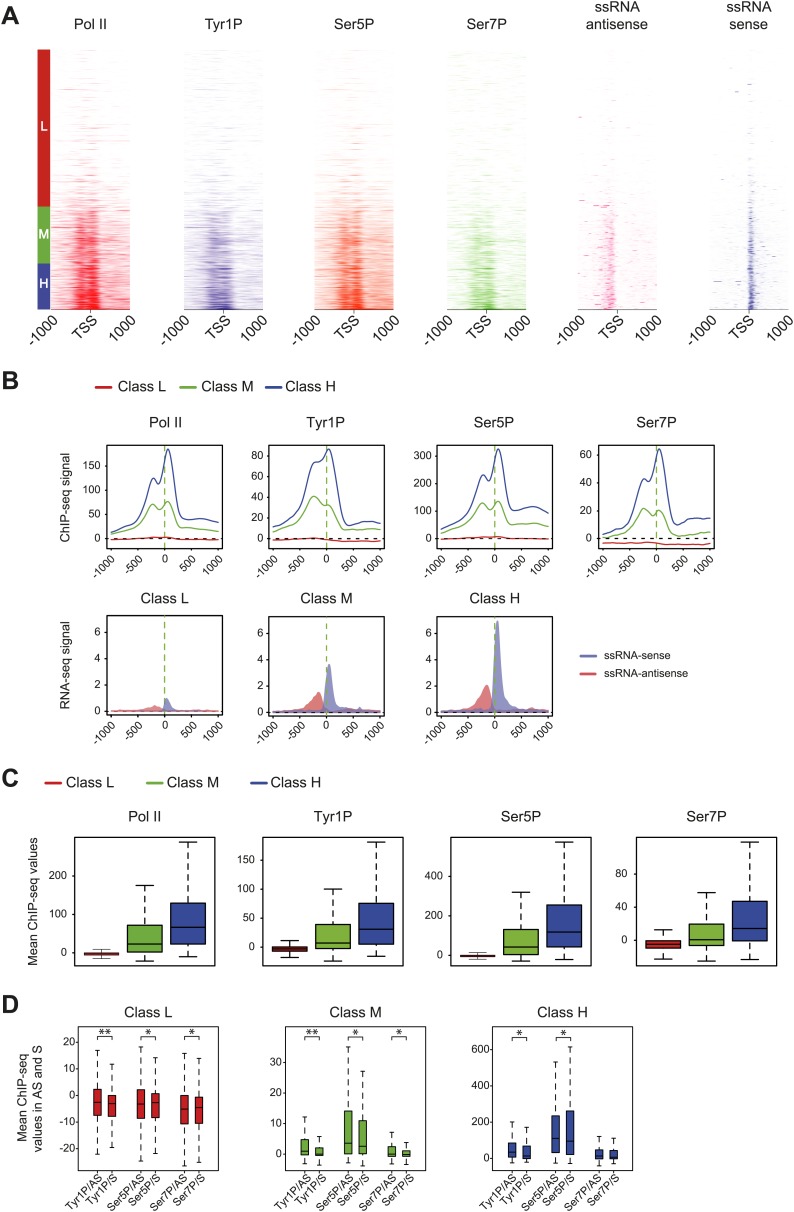
Pol II and CTD PTMs correlate positively with expression. Based on microarray expression data, three groups of genes with low (L, 3414 genes), medium (M, 1238 genes), and high (H, 1007 genes) expression were used to profile Pol II isoforms and short ssRNA at promoters. (**A**) Heatmaps of signal densities for the three defined groups. (**B**) Average profiles of Pol II phospho-isoforms and ssRNA at the three defined groups. (**C**) Boxplots of the mean values retrieved at TSS −/+ 500 bp in the three classes for Pol II (3095, 1169, 957 genes), Tyr1P (3159, 1150, 958 genes), Ser5P (3072, 1157, 956 genes), and Ser7P (3184, 1130, 942 genes). (**D**) Boxplot of regions representing each transcription orientation as in Figure 2E for each class divided by Pol II binding values. p-value (parametric two sided paired *t* test) are respectively: 2.3 × 10^−13^; 5 × 10^−4^; 6 × 10^−3^ (low), 2.4 × 10^−13^; 6 × 10^−3^; 2 × 10^−4^ (medium), 7 × 10^−6^; 0.02; 0.8 (high). Represented number of genes are 3175, 3126, 3074, 3051, 3123, 3134 (low); 1154, 1079, 1154, 1125, 1139, 1084 (medium); 955, 930, 941, 941, 935, 913 (high). **DOI:** http://dx.doi.org/10.7554/eLife.02105.008

**Figure 2—figure supplement 3.**
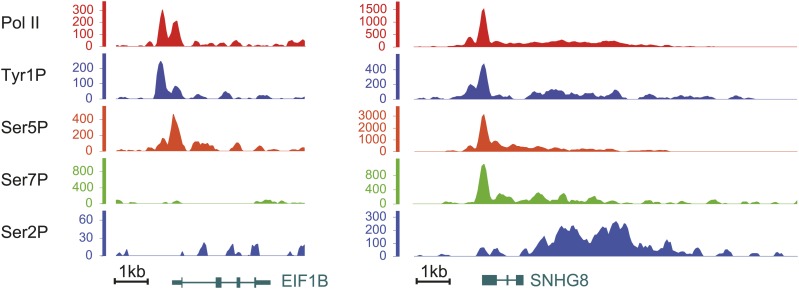
Examples of Tyr1P binding patterns at genic locations. EIF1B and SNHG8 are mainly bound by Tyr1P (3D12) at TSS as for RPL22L1 gene of Figure 2B. **DOI:** http://dx.doi.org/10.7554/eLife.02105.009

**Figure 2—figure supplement 4.**
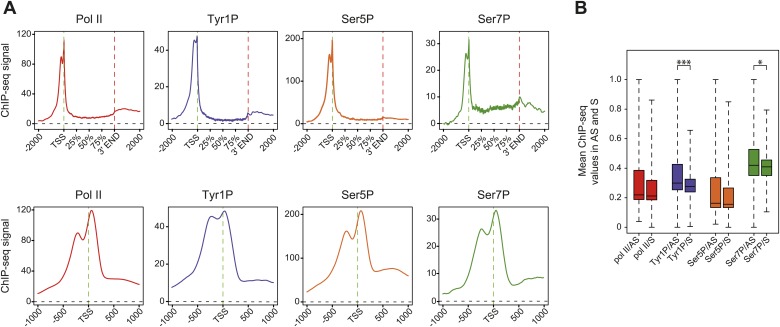
Average profiling of Pol II and phospho-isoforms at genic and promoter locations using wide relaxed threshold selections. (**A**) Composite and TSS focused average profiling of ChIP-seq data as in Figure 2C,D, for a selection threshold of 0 as described in Figure 2—figure supplement 1B, at coding genes locations for Pol II (2714 genes), Tyr1P (3D12, 2987 genes), Ser5P (2697 genes), and Ser7P (3002 genes) in Raji B-cells. (**B**) Boxplots on 4749 genes as in Figure 2E for the less stringent selection showing mean levels of Pol II, Tyr1P, Ser5P, and Ser7P ChIP-seq signal on regions representing each transcription orientation. The p-values (parametric two sided paired *t* test) of the difference of AS vs S signal are Pol II = 0.2, Tyr1p=3.5 × 10^−16^, Ser5p=0.2, Ser7p=0.03. Boxplots do not show outliers for Pol II (3933 genes), Tyr1P (3897 genes), Ser5P (3920 genes), and Ser7P (3878 genes). **DOI:** http://dx.doi.org/10.7554/eLife.02105.010

**Figure 2—figure supplement 5.**
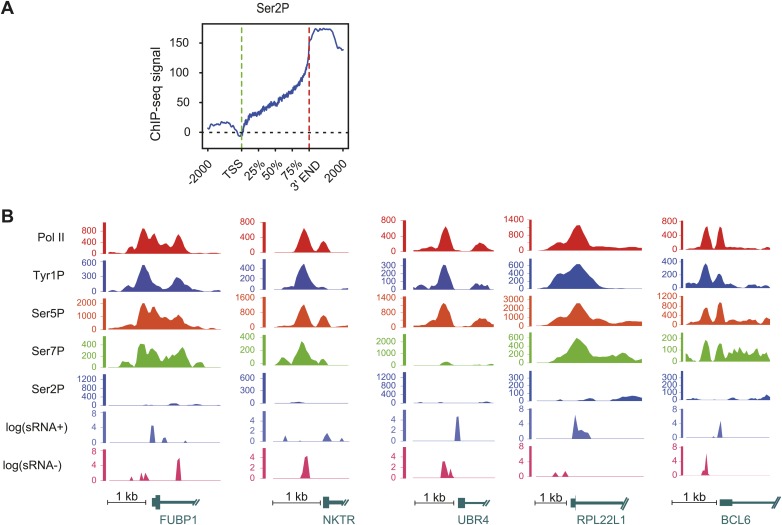
Ser2P average profile at genic locations and examples of Tyr1P signal at promoter locations. (**A**) Ser2P average profile on 1415 genes selected on mean values distribution shown in Figure 2—figure supplement 1B and represented as for Figure 2C. (**B**) Examples of Tyr1P (and other isoforms, short ssRNAs) at promoters of 5 coding genes. These genes show a dominance of Tyr1P (3D12) signal upstream (AS direction) relatively to downstream TSSs and as compared to Pol II and isoforms. **DOI:** http://dx.doi.org/10.7554/eLife.02105.011

**Figure 2—figure supplement 6.**
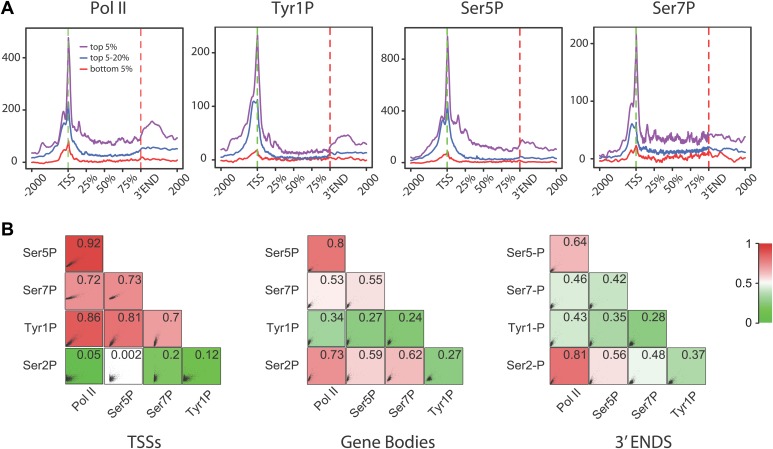
Tyr1P presents a specific pattern of phosphorylation along genes compared to Pol II. (**A**) Genome-wide profiling of Pol II (N20) and CTD isoforms (as in Figure 2) for different classes of binding levels indicate a distribution of Tyr1P more prominent at promoters vs gene bodies as compared to Pol II and Ser7P, but comparable to that of Ser5P. The indicated signal rank of the values is over an area encompassing TSS, GB, and 3′ ends of genes as indicated in the ‘Materials and methods–Gene selection and average binding profiles’. Note that more Tyr1P signal is found at 3′ ends as compared to Ser5P. (**B**) Spearman correlation plots of significantly enriched areas for Pol II and phospho-isoforms (genes size >2 kb) indicate that Tyr1P relates more to Pol II and early transcription marks at promoters than it does at gene bodies or 3′ends. Mean values for Spearman correlation were computed at [TSS-500 bp;TSS+500 bp], [TSS+1000 bp; 3′end-500 bp], and [3′end-500 bp; 3′end+1000 bp] (‘Materials and methods–Correlation of biological replicates and cross-correlation’). **DOI:** http://dx.doi.org/10.7554/eLife.02105.012

**Figure 2—figure supplement 7.**
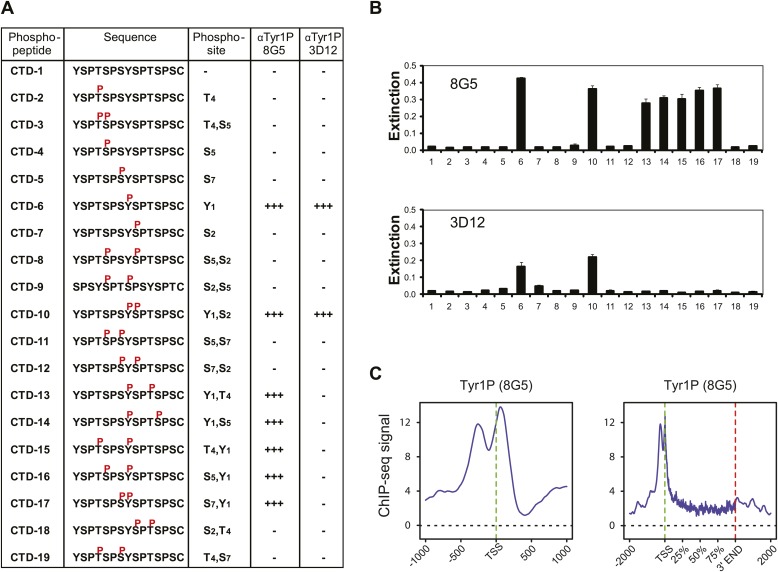
Tyr1P specific antibodies with distinct peptide recognition patterns show similar genome-wide profiling at TSS. (**A**) CTD peptide recognition patterns of 3D12 and 8G5 Tyr1P Abs used in this study. Note that 8G5 shows a wider range of peptide recognition compared to 3D12. (**B**) Specificity and reactivity of mAbs were tested in ELISA experiments towards the peptides CTD-1 to -19. (**C**) Genome-wide profiling of ChIP-seq experiments performed with 8G5 at TSSs (left panel) or at gene body locations on 2365 genes. As for 3D12 Ab, the AS peak is over-represented when compared to Pol II. **DOI:** http://dx.doi.org/10.7554/eLife.02105.013

To assess its relation to transcription genome-wide, we next performed Tyr1P ChIP-seq, using 3D12 mAb, and compared it to Pol II and the other phospho-isoforms. We isolated significantly associated regions based on the signal distribution of the background-subtracted data (Figure 2—figure supplement 1B) and found that Pol II and all isoforms, including Tyr1P, correlated with transcription levels of genes (Figure 2—figure supplement 2). At many gene locations, a predominant signal of Tyr1P at promoters was observed (Figure 2B, Figure 2—figure supplement 3). We further confirmed this by genome-wide profiling of Pol II isoforms at coding-gene locations (Figure 2C, Figure 2—figure supplement 4, Figure 2—figure supplement 5A for Ser2P profile). Our profiling analysis shows that Tyr1P signal is predominantly found at promoters similarly to Ser5P, weak or essentially absent at gene bodies and weak at 3′ends in contrast to Ser2P elongating mark and Ser7P (associated to both promoters and gene bodies). These observations are further supported by quantification of signals at various genic sections (Figure 2—figure supplement 6) and reinforce our conclusion that human Tyr1P is mainly associated to promoters in an early, post-initiation step of transcription. Although we did not further investigate this possibility, in the accompanying manuscript, Hsin et al show that Chicken Tyr1 is found phosphorylated in the nucleoplasm, raising the possibility that Tyr1P is also associated with recruitment of the enzyme and transcription initiation.

Genomic profiling at the vicinity of the transcription start site (TSS) indicates two main peaks of Pol II upstream and downstream of the TSS (Figure 2D, left panel and Figure 2—figure supplement 4). These peaks most likely reflect sense and antisense paused transcription as evidenced by our short strand specific (ssRNA) sequencing analysis, as previously described (Core et al., 2008; Preker et al., 2008; Seila et al., 2008; Fenouil et al., 2012) for mammalian promoters. This transcription results in short promoter-associated transcripts production and might relate to pervasive transcription of promoters in sequence context lacking strong elements imposing directionality. By comparing the signals of these two peaks with that of the Ser5P and Ser7P isoforms, Tyr1P showed a clearly distinct pattern with a more pronounced upstream peak (Figure 2D, Figure 2—figure supplement 4 and examples in Figure 2—figure supplement 5B). We confirmed this result with an independent Tyr1P antibody (8G5) harboring wider range of CTD peptide recognition (Figure 2—figure supplement 7), and by using statistical analysis showing that antisense/sense (AS/S) difference was significant for Tyr1P as compared to other isoforms (Figure 2E, Figure 2—figure supplement 4B). Together, our analyses indicate that Tyr1P is predominantly associated with upstream polymerases, mostly reflecting AS transcription at mammalian promoters.

**Figure 3.**
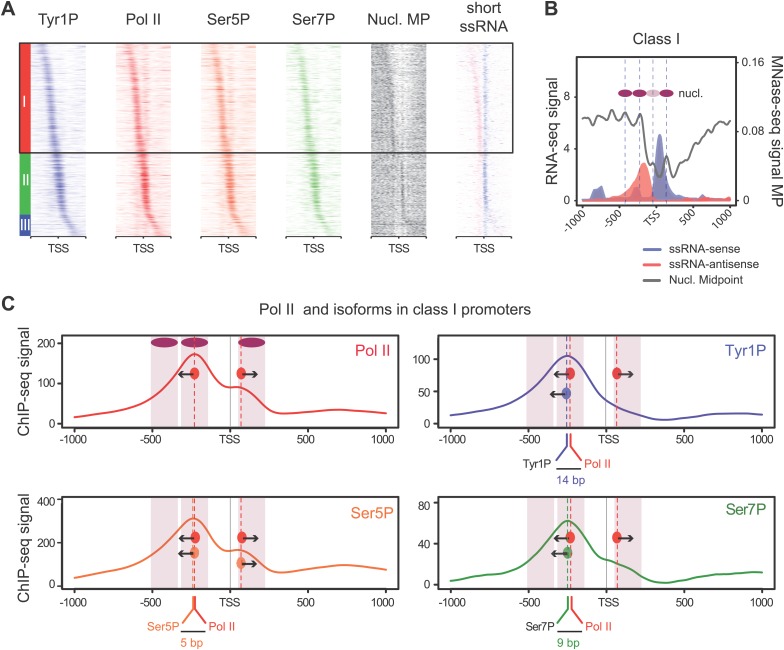
Tyr1 is preferentially phosphorylated in antisense orientation on a particular subset of genes. (**A**) Heatmaps of Tyr1P (3D12), Pol II, Ser5P, Ser7P, nucleosome midpoints (positioning) and short strand specific RNAs (red for AS and blue for S signal) at promoters with a significant level of Pol II. The genes were ordered by position of the main Tyr1P accumulation area from the most 5′ to the most 3′ within −1000 bp and +1000 bp around TSS. Three main classes are defined by Tyr1P occupancy: class I most 5′ (red bar, 1066 genes), class II TSS-proximal (green bar, 579 genes), and class III most 3′ (blue bar, 209 genes). (**B**) Average profiling of short ssRNAs and nucleosomes positions in class I. Positions of the nucleosome midpoints are indicated by a dashed line (nucleosome −3, −2, −1, and +1 from left to right). (**C**) Profiles of Pol II and CTD isoforms in class I. Red, blue, orange, and green dashed lines indicate the average position of the maximum values of Pol II, Tyr1P (3D12), Ser5P, and Ser7P signals, respectively. The distance between Pol II leading edge and isoforms is indicated below each graph. The borders of nucleosomes −3, −2, and +1 (from left to right) are shown as pink rectangles whereas the red, blue, orange, and green circles represent Pol II, Tyr1P, Ser5P, and Ser7P, respectively with indication of directionality based on the short ssRNA signals. **DOI:** http://dx.doi.org/10.7554/eLife.02105.014

We previously showed that mammalian promoters associated with Pol II can be grouped in three main classes in mouse T-lymphocytes (Fenouil et al., 2012), based on ranking of the main Pol II signal from the most upstream to the most downstream of the TSS. We reproduced this result and the main features of the three groups in human Raji B-cells by ranking the signal of Tyr1P (Figure 3A, Figure 3—figure supplement 1A,B). The first class (the majority of genes), with Pol II signals most upstream of TSSs, harbors strongly paused Pol II at promoters with high GC content and CpG islands (CGIs) and is associated with the highest level of bidirectional and AS transcription. The second class, with a sharper Pol II peak centered close to the TSS and lower GC content, contains mostly mono-directional sense paused transcription whereas the third class contains more downstream Pol II with less pause. We then focused our attention on class I that contains most AS short RNAs. In this class, Tyr1P is essentially observed in AS while Ser5P, Ser7P, or total Pol II generally show a second peak around the TSS reflecting sense and therefore bidirectional transcription (Figure 3C, Figure 3—figure supplement 1C). This indicates that AS Tyr1P relates to one specific class of promoters and suggests that in AS orientation, Tyr1P associates mainly with the leading edge of Pol II. Pleading for this hypothesis, the location of the AS Tyr1P in class I is found more downstream on average as compared to Pol II or Ser5P, and locates just after the −2 nucleosome midpoint (Figure 3B,C). A more detailed investigation of the individual positions of phospho-isoforms further shows that for the majority of promoters significantly associated with AS short RNAs in class I, Tyr1P is either located at the immediate proximity or after the main Pol II peak (Figure 3—figure supplement 2) suggesting that it might play a role in early elongation. Although Ser7P displayed similar characteristics, its influence on transcription of coding genes is likely to be minor, as Ser7 mutations do not show significant phenotype (Chapman et al., 2007) or transcriptome impairment (JCA and DE, unpublished observations). We overall conclude that Tyr1P is a CTD PTM that associates with the 5′ end of genes and shows a stronger linkage to paused Pol II at promoters with bidirectional and AS transcription.

**Figure 3—figure supplement 1.**
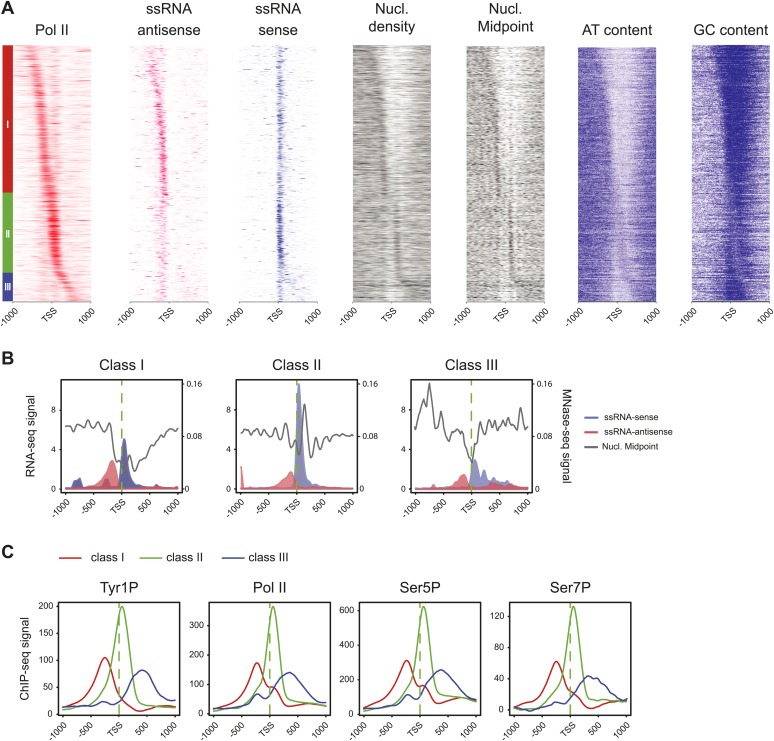
Three classes of Pol II-bound promoters ordered by Tyr1P location in human Raji cells. (**A**) Heatmaps of a selection of Pol II-bound promoters for ssRNAs, nucleosome and AT, GC contents ordered by Tyr1P (3D12) maximum signal from the most upstream to the most downstream of the annotated TSSs (as previously described in mouse lymphocytes, Fenouil et al., 2012). Note that Pol II main accumulation areas occur at proximity of the main nucleosome position for each promoter class. As described before (Fenouil et al., 2012), GC content and CpG islands correlate with nucleosome depletion. (**B**) Profiles of ssRNAs (sense and antisense) and nucleosome in the three groups. (**C**) Profiles of Pol II and CTD isoforms in the three classes of promoters as indicated. **DOI:** http://dx.doi.org/10.7554/eLife.02105.015

**Figure 3—figure supplement 2.**
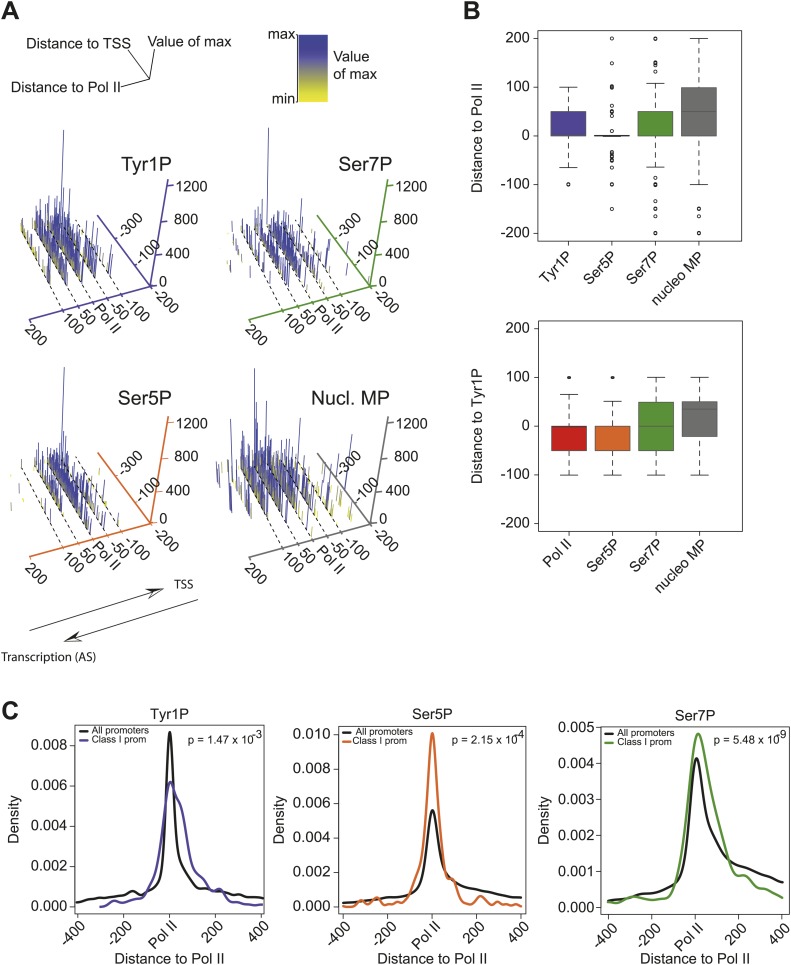
CTD isoforms and nucleosome distribution around Pol II upstream of TSSs in class I promoters. (**A**) 3D plots of Tyr1P, Ser5P, Ser7P and nucleosomes midpoints (MP) maximum signal locations as compared to Pol II ChIP-seq maxima for genes of group 1 of Figure 3A. Only genes with a significant signal of antisense ssRNA and higher than sense ssRNA were taken into account (see ‘Materials and methods–CTD isoforms and nucleosomes midpoint maximal peaks spatial organization analysis’ for details). The positive values of the distance to Pol II axis (in bp) indicate that maximum signals are located after Pol II in opposite direction of TSSs whereas negative values are in the inverse orientation. The number of maximal peaks before, after or colocalized with Pol II for Tyr1P, Ser5P, and Ser7P are 90/265/174, 99/152/278, 125/234/170, respectively. Note that most of the Tyr1P max values are located after Pol II whereas Ser5P is mainly found around Pol II main signal. (**B**) 2D Boxplots of the maximum values shown in (**A**) (upper panel) and for an independent analysis using Tyr1P max signal as reference (lower panel). In both cases Tyr1P locates at or after the leading edge of Pol II. (**C**) Distance to Pol II distribution of Tyr1P, Ser5P, and Ser7P for class I promoters selected as described in (**A**). Data is represented in bins of 10 (‘Materials and methods–Processing of sequenced tags’). The difference of distribution with the whole set of genes (black line) was assessed by a nonparametric Kolmogorov-Smirnov test. p-values are indicated at the top-right of each panel. **DOI:** http://dx.doi.org/10.7554/eLife.02105.016

**Figure 4.**
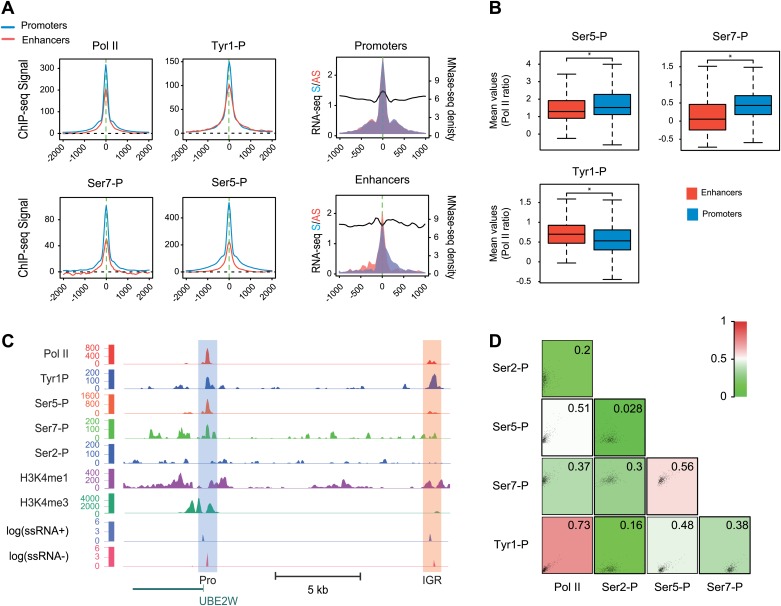
Tyr1P is a hallmark of enhancers relative to Pol II and promoters signal. (**A**) Average profiling of Pol II, Tyr1P (3D12), Ser5P, Ser7P, nucleosomes occupancy, and short ssRNAs. 390 active putative enhancers (red) and 4618 control promoters (blue) were detected in human Raji B-cells (‘Materials and methods–Selection of enhancers and promoters using Pol II’). Profiles are centered on Pol II ChIP-seq maximal signal and are not oriented. (**B**) Boxplots of mean ChIP-seq signal on selected enhancer and control promoter regions for Ser5P (371/4378 values), Ser7P (368/4257 values), and Tyr1P (372/4266 values). Signals were normalized by the mean ChIP-seq signal of Pol II on the same regions. All marks show a significant difference (nonparametric Mann-Whitney-Wilcoxon test, p-values <10^−10^). (**C**) Example of Tyr1P at promoter and putative enhancer. (**D**) Spearman cross-correlation between Pol II, Ser5P, Ser7P, Ser2P, and Tyr1P (3D12) at intergenic putative enhancers. Tyr1P and Pol II best correlate with each other. **DOI:** http://dx.doi.org/10.7554/eLife.02105.017

Many groups including ours have shown that highly active and tissue-specific enhancers are transcribed by Pol II in various tissues (De Santa et al., 2010; Kim et al., 2010; Koch et al., 2011; Natoli and Andrau, 2012). These enhancers can also be hallmarked by the occurrence of H3K4me1^high^/H3K4me3^low^ epigenetic marks combination (Koch et al., 2011; Pekowska et al., 2011). To investigate if Tyr1P can be detected at enhancers, we first isolated intergenic regions (IGRs) with stringent criteria in B-cells using Pol II, H3K4me1, and me3 signals. These were further discriminated from noncoding promoters using the relative ratio of H3K4me1/me3 (Pekowska et al., 2011; Li et al., 2012), and from both unannotated coding and some long intergenic noncoding genes using the absence of H3K36me3 that marks gene bodies (Guttman et al., 2009). Using these criteria, we isolated 390 B-cells enhancers (Figure 4—figure supplement 1A–D). Our selection was further validated using tissue-specificity analyses (Figure 4—figure supplement 1E) indicating IGRs associated with genes specific to B-cells. We next performed profiling of the various Pol II isoforms at these enhancers. As before (Koch et al., 2011), we observed that these IGRs were associated with Ser5P (Figure 4A) but not with Ser2P Pol II (not shown) as well as with short transcripts (reflecting paused transcription) and a discrete nucleosome depleted region. Consistent with early elongating Pol II at enhancers, we found signal for both Ser7P and Tyr1P at these IGRs. Importantly, Tyr1P appeared more bound to enhancers as compared to promoters and total Pol II (Figure 4B,C, Figure 4—figure supplement 2), suggesting that Tyr1 is more phosphorylated than Ser5 or Ser7 at enhancers and represent a hallmark of these essential areas of the genome. Additionally, Tyr1P also displayed the best correlation with Pol II at isolated enhancers (Figure 4D). Finally, using an independent selection for active enhancers based on H3K27ac brought very similar results (Figure 4—figure supplement 3). Together, our investigations showed that Tyr1P is a strong signature of Pol II-transcribed active enhancers associated with tissue-specific gene expression.

**Figure 4—figure supplement 1.**
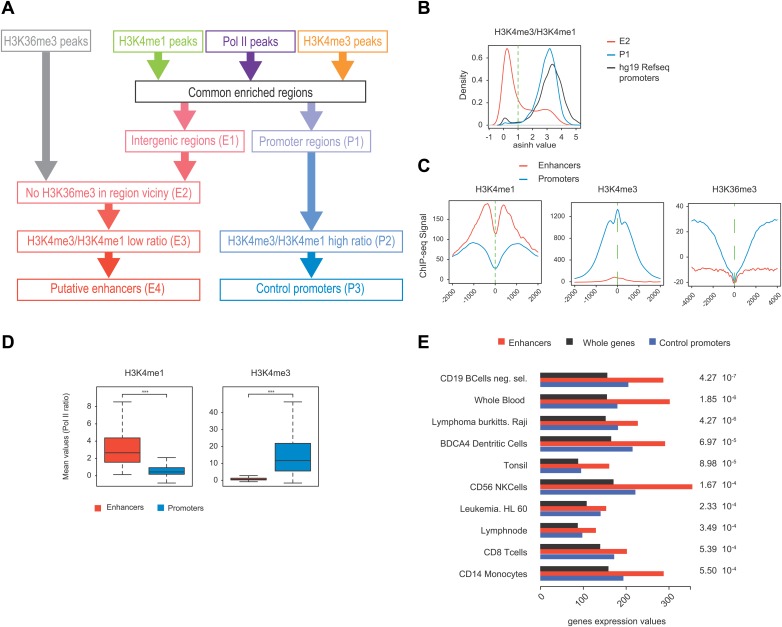
Pol II-bound enhancer selection procedure and features. (**A**) Workflow of the enhancers (390) and control promoters (4618) selection based on ChIP-seq of H3K36me3, H3K4me3, H3K4me1, and Pol II. Details of procedure and number of regions isolated at each step (E1-4 and P1-3) are indicated in ‘Materials and methods–Selection of enhancers and promoters using Pol II’. (**B**) Plot of H3K4me3/me1 mean values ratios of selected intergenic regions at step E2 (in red), promoter regions at step P1 (in blue), and Hg19 RefSeq annotated promoters (in black). To stringently select isolated promoters and intergenic regions shown in (**A**) and attribute their putative enhancer and control promoter status, a threshold was defined (in dashed green line). (**C**) Nonoriented profiling of epigenetic marks associated with putative enhancers (in red) and control promoters (in blue) selected at steps P3 and E4 of procedure described in (**A**) and centered on the main Pol II peak as in Figure 4A. (**D**) Boxplots of H3K4me3 (363/4325 genes plotted) and H3K4me1 (375/4259 genes plotted) signals at putative enhancers (in red) and control promoters (in blue). Nonparametric Mann-Whitney-Wilcoxon test gave p-values <10^−152^. (**E**) Tissue specificity analysis of the genes associated with putative enhancers (closest genes on each side of the isolated genomic loci) compared to genes of HGU133 array (whole genes, see ‘Materials and methods–Tissue specificity analysis’). The isolated tissues are ranked by p-values (indicated on the left) from top to bottom. This analysis indicates that both WT (CD19) and Raji human B-cells are among the most significant tissues thus validating the putative enhancer regions identified in our analysis and as described in mouse lymphocytes (Li et al., 2012). **DOI:** http://dx.doi.org/10.7554/eLife.02105.018

**Figure 4—figure supplement 2.**
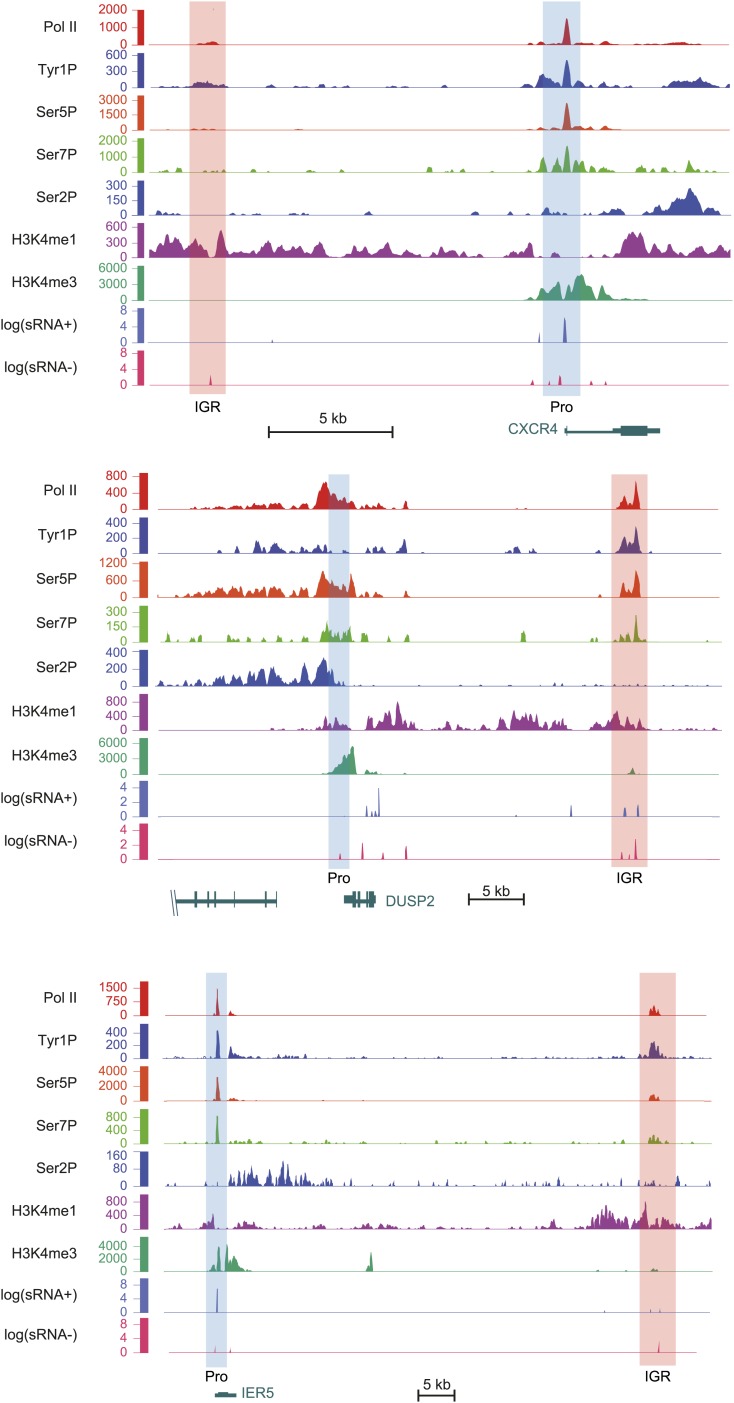
Examples of Tyr1P enhancer association upstream or downstream of CXCR4, DUSP2, and IER5 genes. As in Figure 4, light orange and blue rectangles highlight enhancer and promoter locations with higher H3K4me3 at promoters and higher H3K4me1 at enhancers. Relative amount of Tyr1P is higher at enhancers as compared to Pol II and to promoters. H3K4me3 level at CXCR4 IGR was observed but is not visible due to the scale used and because of high level of signal at promoter. **DOI:** http://dx.doi.org/10.7554/eLife.02105.019

**Figure 4—figure supplement 3.**
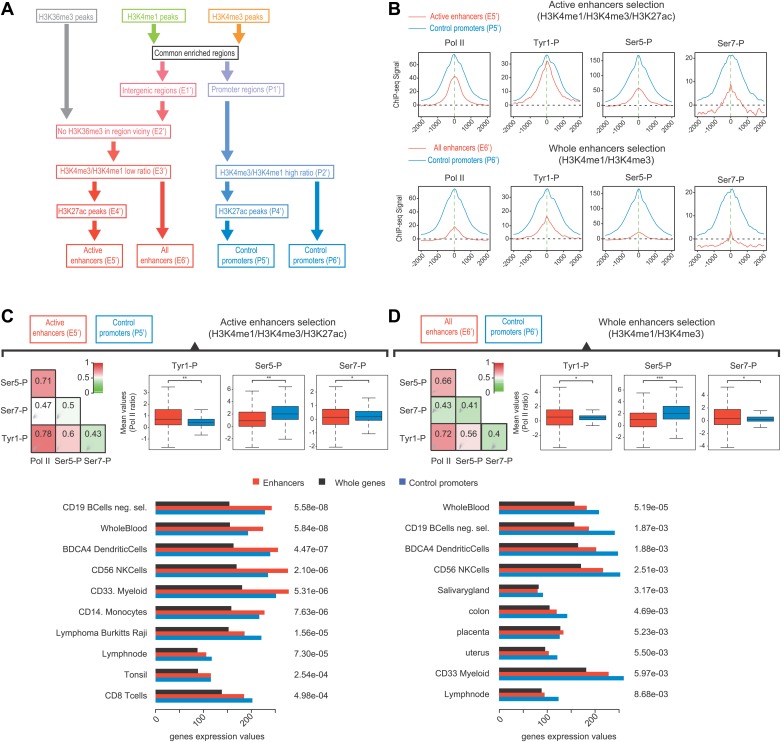
Selection of enhancers using H3K27ac also shows a dominance of Tyr1P on active and tissue specific enhancers. (**A**) Workflow of a complementary selection of enhancers (927/2598 active), and control promoters (5946/6057 active) based on ChIP-seq of H3K36me3, H3K4me3, and H3K4me1. H3K27ac was used to extract specifically active enhancers from the whole set. Details of procedure and number of regions isolated at each step (E1'-6' and P1'-6') are indicated in ‘Materials and method–Selection of active enhancers and promoters using H3K27ac’. (**B**) Average profiles of Pol II and isoforms for active enhancers/promoters and the whole set of enhancers/promoters. (**C**) Active (H3K27ac selection) enhancers show increased enrichment over Pol II and tissue-specific gene expression. As in Figure 4, Spearman correlation, boxplots of comparison of levels of Pol II isoforms, and tissue specificity analyses indicate Tyr1P to be over-enriched at active enhancers as compared to Pol II and promoters. Nonparametric two-sided Mann-Whitney-Wilcoxon test for boxplots of Ser5P (780/5068 values), Ser7P (752/4953 values), and Tyr1P (739/5233 values) yields p-values of 5.1 × 10^−56^, 7.05 × 10^−4^, and 2.1 × 10^−30^, respectively. (**D**) Whole enhancer set (H3K4me1/3) analysis as in (**C**). Nonparametric two sided Mann-Whitney-Wilcoxon test for boxplots of Ser5P (2220/5141 values), Ser7P (2186/5005 values), and Tyr1P (2112/5306 values) yields p-values of 2.6 × 10^−139^, 6.8 × 10^−4^, and 8.09 × 10^−4^, respectively. **DOI:** http://dx.doi.org/10.7554/eLife.02105.020

Here, we described that Tyr1P associates with 5′ Pol II and AS transcription at promoters and is a signature of active, tissue-specific enhancers in human B-cells. These findings contrast with features of Tyr1P in yeast, which is located at gene bodies and proposed to play a role in elongation by impairing termination factor recruitment (Mayer et al., 2012). These apparent discrepancies thus provide an interesting paradigm whereby a conserved PTM has evolved to display specialized functions specific to metazoans. However, *S. cerevisiae* genes are very compact, mostly devoid of introns and promoters structure is also extremely divergent in both length and sequence between yeast (around 100–200 nt, AT-rich) and mammals (around 1000 nt, GC-rich). Furthermore, enhancers do not exist per se in yeast. In an accompanying manuscript, Hsin et al. (2014) describe similar observations regarding stability of Y1F mutant in chicken cells and involvement of Tyr1P in AS transcription at promoters, thus providing further evidence that our observations are conserved in vertebrates. We therefore speculate that differential CTD PTMs might not only reflect, but also play a role in regulating the directionality of transcription. How would Tyr1P behave in organisms with less prominent bidirectional transcription at promoters such as *Drosophila* (Core et al., 2012) thus represents an interesting evolutionary question to be addressed in future studies.

Based on the spatial location of Tyr1P in class I promoters, mostly found at the leading edge of Pol II in opposite orientation of the gene, it is tempting to speculate that this PTM might be involved in a transcriptional state marking the transition between early and productive elongation, providing a checkpoint for transcriptional complexes to proceed in productive elongation. Depending on the level of Tyr1P at promoters, Pol II might become competent for elongation as well as for overcoming the nucleosomal barrier both in sense and antisense orientation. Since less Pol II molecules are able to effectively enter elongation in AS orientation, more accumulation of the Tyr1P could be observed upstream of the TSS toward the leading edge of Pol II. This could also explain degradation of Y1F mutant that is due to absence of Tyr1P checkpoint signal, would accumulate around the edge of the promoters and become degraded. Finally there could also be a link between hyperphosphorylation of Tyr1 in AS orientation and exosome machinery recruitment to degrade nascent RNA prior release of the Pol II enzyme (Preker et al., 2008; Andersen et al., 2013). We believe our work will thus provide a new frame of investigation to decipher the complexity of mechanisms leading to transcriptional activation, at the heart of gene regulation.

## Material and methods

### Antibodies

Generation and validation of modification specific mAbs have been described before: Tyr1P mAb (3D12, Mayer et al., 2012) and 8G5 (see Figure 2—figure supplement 7), Ser2P (3E10), Ser5P (3E8), and Ser7P (4E12, Chapman et al., 2007), Thr4P (6D7, Hintermair et al., 2012).

For further characterization of specificity, the 3D12 and 8G5 Tyr1P antibodies were analyzed in ELISA experiments using CTD-like peptides with different modification patterns (Peptide Specialty Laboratories GmbH, Heidelberg, Germany) coupled to 96-well maleimide plates (Thermo Fisher Scientific Inc., Rockford, IL USA) as antigen (Figure 2—figure supplement 7). Peptides were incubated with the monoclonal antibodies and biotinylated, subclass-specific antibodies, respectively. After incubation with horseradish peroxidase (HRP)-coupled avidin, H_2_O_2_ and TMB (3,3',5,5'-tetramethylbenzidine) were added. Absorbance of each well was measured at 650 nm after color change and quantitated with an ELISA reader.

### Extracts, western blots, and co-immunoprecipitation

#### Immunoprecipitation (IP) experiments

3 × 10^6^ Raji cells were lysed in 200 µl IP buffer (50 mM Tris–HCl, pH 8.0, 150 mM NaCl, 1% NP-40 (Roche, Germany), 1x PhosSTOP (Roche), 1x protease inhibitor cocktail (Roche)) for 20 min on ice. All samples were sonicated on ice using a BRANSON Sonifier 250 (15 s on, 15 s off, 50% duty) and centrifuged at 14,500 rpm for 15 min at 4°C. The supernatant was incubated with antibody-coupled protein G/A-sepharose (1:1) beads (2.5 µg of antibodies for 4 hr at 4°C, followed by two washes with 1 ml IP buffer) rotating overnight. Beads were washed several times with 1 ml IP buffer and proteins were boiled off Sepharose beads in Laemmli buffer containing 8M urea for SDS-Page.

#### Western blots

Samples of protein were harvested following treatment using 2x Laemmli buffer. Protein equivalent to 200,000 cells was loaded in 20 µl Laemmli, per lane, and subjected to SDS-PAGE on a 6.5% gel before transfer to nitrocellulose (GE Healthcare, Germany). Membranes were either stained with affinity purified, IR-labelled secondary antibodies against rat (680 nm; Alexa, Invitrogen) and mouse (800 nm; Rockford, Biomol), and revealed using the Odyssey (Licor), or stained with hrp-conjugated secondary antibodies against rat (Sigma), mouse (Promega), or rabbit (Promega), and revealed by enhanced chemiluminescence.

### Generation and analysis of Y1F mutant

#### Construction of the CTD Y1F mutant

Construction of wild-type and mutant (Y1F) Rpb1 expression vectors was performed as follow: The DNA sequence of RPB1 CTD comprising amino acids of repeats 1–52 (aa 1593–1970) was synthesized and cloned into a vector LSmock (Chapman et al., 2004) and recombinant HA-tagged wild-type RPB1 was established. Alternatively, a CTD DNA sequence was synthesized with a replacement of amino acid tyrosine to phenylalanine in repeats 4 to 51 of CTD. Both vectors were sequenced before usage.

#### α-Amanitin resistant cell lines and cell culture

Raji is an Epstein-Barr-virus-positive Burkitt's lymphoma cell line. Cells were transfected with the wild-type and (Y1F) Rpb1 expression vectors by electroporation (10 μg of plasmid DNA/10^7^ cells; 960 μF, 250 V). Polyclonal cell batches were established after selection with G418 (1 mg/ml) for 10–12 days. Expression of recombinant Rpb1 was induced by removal of doxocyclin. 24 hr after induction, cells were cultured in the presence of 2 μg/ml α-amanitin (Sigma). Cells were grown in RPMI 1640 medium supplemented with 10% fetal calf serum, 1% penicillin streptomycin (GIBCO; Invitrogen, Germany), and 2 mM L-glutamine (GIBCO; Invitrogen) at densities between 2 × 10^5^ to 10^6^ cells/ml.

### ChIP-seq, MNase-seq, and RNA-seq experiments

ChIP-seq and MNase-seq experiments were performed essentially as described before using same standard and QC for experiments (Fenouil et al., 2012). Experimental details of individual experiments, including replicates when applicable, are also indicated in Table 1.

**Table 1.** Summary of ChIP conditions and bioinformatics treatment for each experiment (NR = not relevant, NA = not available) **DOI:** http://dx.doi.org/10.7554/eLife.02105.021

	ChIP antibodies and conditions used (* For ChIP-QPCR)											Peak detection	
Experiment	Antibody (clone)	Origin	Reference Antibody	Number of cells	Antibody/Beads	Washes (RIPA/TE)	Replicates Number	Tags Not Aligned/Multiple Alignment (× 10^6^)	Tags Used (× 10^6^)	Lanes Number	Extension Size (bp)	Threshold	Max Gap
Pol II	Total (N-20)	Rabbit polyclonal	Santa Cruz (sc-899x)	1 × 10^8^	20 µg/200 µl	8x/1x	1	8.93	19.83	1	176	80	350
							2	17.94	33.02	2	166		
							3	16.54	28.48	1	156		
H3K4me1	H3K4me1	Rabbit polyclonal	Abcam (ab8895)	5 × 10^6^	2 µg/20 µl	8x/1x	1	9.35	7.83	1	176	60	700
							2	7.59	20.93	1	226		
H3K4me3	H3K4me3	Rabbit polyclonal	Abcam (ab8580)	5 × 10^6^	2 µg/20 µl	8x/1x	1	7.12	2.61	1	186	50	400
							2	NA	14.14	1	123		
H3K36me3	H3K36me3	Rabbit polyclonal	Abcam (ab9050)	2 × 10^7^	8 µg/80 µl	8x/1x	1	NA	21.21	1	196	40	1000
							2	NA	5.57	1	316		
H3K27ac	H3K27ac	Rabbit polyclonal	Ab4729	5 × 10^6^	2 µg/20 µl	5x/1x	1	5.33	52.50	1	197	100	750
Tyr1P	Tyr1P (3D12)	Rat monoclonal	Mayer et al. (2012)	1 × 10^8^	10 µg/100 µl	5x/1x	1	12.30	15.56	1	206	NR	NR
							2	9.98	15.55	1	276		
	Tyr1P (8G5)	Rat monoclonal	This article	1 × 10^8^	10 µg/100 µl	5x/1x	1	30.26	28.78	1	187	NR	NR
Ser2P	Ser2P (3E10)	Rat monoclonal	Chapman et al. (2007)	2 × 10^8^	80 µg/400 µl	5x/1x	1	9.31	11.28	1	192	NR	NR
							2	9.85	15.94	1	286		
Ser5P	Ser5P (3E8)	Rat monoclonal	Chapman et al. (2007)	1.2 × 10^8^ (2.5 × 10^7^*)	24 µg/240 µl (5 µg/50 µl*)	8x/1x	1	NA	13.98	1	146	NR	NR
							2	NA	3.57	1	216		
Ser7P	Ser7P (4E12)	Rat monoclonal	Chapman et al. (2007)	1 × 10^8^	10 µg/100 µl	5x/1x	1	NA	16.46	1	156	NR	NR
							2	NA	1.92	1	226		
Short-RNA-seq	NR	NR	NR	1 × 10^7^	NR	NR	1	NA	9.87	1	NR	NR	NR
MNase-seq	NR	NR	NR	2 × 10^7^	NR	NR	1	90.00	289.60	1	152/NR midpoints**	NR	NR
Input	NR	NR	NR	NR	NR	NR	1	20.10	18.18	1	126	NR	NR
							2	NA	29.74	1	146		
							3	15.41	24.93	1	118		
							4	11.20	28.32	1	196		

** For MNase-seq, the experiment was performed and processed in pair-end. For nucleosome density, tags were not elongated but connected and the indicated sequence average length is withdrawn by our analysis pipeline using the pair-end information. For midpoints analyses, elongation does not apply and data treatment is indicated earlier in ‘Materials and methods–Processing of sequenced tags’.

#### ChIP-seq

Briefly, for ChIP-seq experiments, Raji B-cells were directly crosslinked in 25 ml of growth medium. Crosslinking was performed with the addition of 1/10th volume of crosslinking solution (11% formaldehyde, 100 mM NaCl, 1 mM EDTA pH 8, 0.5 mM EGTA pH 8, 50 mM Hepes pH 7.8) for a final formaldehyde concentration of 1% for 10 min at room temperature. The reaction was quenched with the addition of 250 mM glycine and incubation at room temperature for 5 min. Cells were washed twice with cold DPBS and counted. All buffers contained EDTA-free protease inhibitor cocktail (Roche, France) and phosphatase inhibitors (Thermo Scientific, France) to final concentration of 1x together with 0.2 mM PMSF and 1 µg/ml pepstatin. 5 × 10^7^ cells were then lysed in 2.5 mL LB1 buffer (50 mM Hepes pH 7.5, 140 mM NaCl, 1 mM EDTA pH 8, 10% glycerol, 0.75% NP-40, 0.25% Triton X-100) by incubation at 4°C for 20 min. Nuclei were collected by centrifugation at 1350×*g* and washed in 2.5 ml LB2 (200 mM NaCl, 1 mM EDTA pH 8, 0.5 mM EGTA pH 8, 10 mM Tris pH 8) for 10 min. Nuclei were then centrifuged and resuspended in 1.5 ml of LB3 (1 mM EDTA pH 8, 0.5 mM EGTA pH 8, 10 mM Tris pH 8, 100 mM NaCl, 0.1% Na-Deoxycholate, 0.5% N-lauroylsarcosine) and sonicated using a Misonix 4000 (Misonix Inc, Farmingdale, NY, USA) sonicator for 14 cycles of 30 s ON and 30 s OFF at amplitude of 40. After sonication Triton X-100 was added to 1% final concentration and cellular debris was precipitated by centrifugation at 20000×*g* for 10 min in a refrigerated centrifuge. Aliquots of clear supernatant were transferred to new tubes, snap frozen, and kept at −80°C until use. 50 µl aliquots were taken to serve as input control as well as sonication quality control.

Inputs were combined with an equal volume of 2x elution buffer (100 mM Tris pH 8, 20 mM EDTA pH 8, 2% SDS) and incubated overnight in a water bath at 65°C for 13–15 hr. SDS was then diluted by the addition of an equal volume of TE (10 mM Tris pH 8, 1 mM EDTA pH 8) and RNA was digested by RNase A at a final concentration of 0.2 μg/ml at 37°C for 2 hr. Samples were subsequently Proteinase K treated at 55°C for 2 hr at a final concentration of 0.2 μg/ml. DNA was purified by two subsequent phenol:chloroform:isoamylalcohol (25:24:1, pH 8) extractions and followed by a Qiaquick purification (PCR purification columns, Qiagen, Germany). DNA concentration was measured using a Nanodrop 1000 (Thermo Scientific, France) and 4 ng DNA was analyzed using High Sensitivity DNA chips on a 2100 Bioanalyzer to verify sonication efficiencies.

All experiments were performed using Dynabeads (Invitrogen, USA) coated with Protein-G. Beads were washed 3x with 1 ml and subsequently resuspended in 250 μl of blocking solution (0.5% BSA in 1x DPBS). After the addition of the antibody, the beads were incubated at 4°C overnight on a rotating wheel. Unbound antibodies were removed through three further washes with 1 ml of blocking solution. Beads were resuspended in 100 μl of blocking solution, chromatin extracts were added, and the mix was incubated overnight at 4°C on a rotating wheel.

EDTA-free protease inhibitors (Roche) were added to all washing buffers to a final concentration of 1x together with 0.2 mM PMSF and 1 μg/ml pepstatin. Beads were washed 8 times in RIPA buffer (50 mM Hepes pH 7.6, 500 mM LiCl, 1 mM EDTA pH 8, 1% NP-40, 0.7% Na-Deoxycholate) and once in TE+ (10 mM Tris pH 8, 1 mM EDTA pH 8, 50 mM NaCl). Immunoprecipitated chromatin was recovered from the beads with two subsequent elution steps at 65°C for 15 and 10 min in 110 μl and 100 μl of elution buffer (50 mM Tris pH 8, 10 mM EDTA pH 8, 1% SDS), respectively. The two eluates were combined and incubated at 65°C overnight (13–15 hr) for crosslink reversal. DNA was purified as described for the input (see Table 1 for a summary of ChIP conditions for each experiment).

Prior to sequencing, ChIP DNA was quantified using the double stranded DNA HS kit on a Qubit apparatus (Life Technologies, USA) and 1 ng ChIP DNA was analyzed on a High Sensitivity DNA chip on a 2100 Bioanalyzer (Agilent Technologies, USA). DNA yields were typically between 5 and 100 ng for different antibodies. At least 1 ng of ChIP or input DNA was used for library preparation according to the Illumina ChIP-seq protocol. After end-repair and adapter ligation, fragments were size-selected (cut) on an agarose gel prior to pre-amplification and clustering. The size-selected and pre-amplified fragments were verified on a 2100 Bioanalyzer (Agilent Technologies, USA) before clustering and sequencing on a Genome Analyzer II or HighSeq 2000 (Illumina, USA) according to manufacturer's instructions.

#### MNase-seq

For sequencing of nucleosomal DNA, 2 × 10^7^ cells were resuspended in 50 μl Solution I (150 mM sucrose, 80 mM KCl, 5 mM K_2_HPO_4_, 5 mM MgCl_2_, 0.5 mM CaCl_2_, 35 mM HEPES pH 7.4) and NP40 was added to a final concentration of 0.2%. Cell membranes were permeabilized for one minute at 37°C. For nucleosomal digestion, 40U of MNase was added with 0.5 ml of Solution II (150 mM sucrose, 50 mM Tris pH 8, 50 mM NaCl, 2 mM CaCl_2_) and incubated for 30 min at room temperature. The reactions were stopped with the addition of EDTA to a final concentration of 10 mM. The cells were lyzed using 1.45 ml of SDS Lysis Buffer (1% SDS, 10 mM EDTA pH 8, 50 mM Tris pH 8), with a 10 min incubation at 4°C. A 200 μl aliquot was taken for purification and the remaining extract was stored at −80°C. An equal volume of TE (200 μl) was added to the aliquot, followed by subsequent 2 hr treatments with each 0.2 μg/ml final concentrations of RNase A and Proteinase K at 37°C and 55°C, respectively. DNA was extracted by two subsequent phenol:chloroform:isoamylalcohol (25:24:1) extractions, further purified using QIAquick PCR purification columns (Qiagen, Germany) and eluted in 50 μl of water. The quality of nucleosomal digestion was verified by running 2 ng of DNA on High-Sensitivity 2100 Bioanalyzer chips (Agilent, USA). The typical Bioanalyser profile in our standardized conditions shows a clear peak of mononucleosome at 146 −/+ 5 bp that represents 80% of the nucleosomal DNA (the rest of the digested material spreads essentially in di- and tri-nucleosomal DNA). After library preparation, DNA fragments corresponding to mononucleosomes were cut from an agarose gel and subsequently clustered and sequenced on Genome Analyzer II (Illumina, USA) according to manufacturer's instructions.

#### Short strand specific RNA-seq

Total RNA was extracted from 1 × 10^7^ Raji cells using TRIzol (Life Technologies, USA) according to the manufacturer's instructions with some modifications to ensure higher recovery rates of small RNAs. This was achieved by addition of 10 μg of linear acrylamide (Life Technologies, USA) before RNA precipitation. DNA was digested using the rigorous Turbo DNase (Ambion, USA) treatment as per manufacturer's instructions. RNA quantity was measured on a Qubit apparatus (Life Technologies, USA) using RNA assay kit and the quality was verified using RNA pico chips on a 2100 Bioanalyzer (Agilent Technologies, USA).

Before preparation of sequencing libraries, small RNAs were enriched from 10 µg total RNA by using mirVana RNA Isolation kit (Life Technologies, USA) using manufacturer's protocol for small RNA enrichment. Strand specific RNA-seq library was constructed with ScriptMiner Small RNA-seq Library Preparation Kit (Epicenter, USA) according to manufacturer's recommended protocol. Briefly, after both 5′ and 3′ adapter ligation, resulting cDNA library was PCR amplified with 14 amplification cycles. Purified library DNA was run on a 10% TBE-PAGE gel and library DNA corresponding to transcripts between 15 nt and 50 nt was cut from the gel and transferred into 0.5 ml tubes with punctured bottoms which were in turn placed in 2 ml collection tubes. Gel slices were crushed into 2 ml tubes by a 2 min centrifugation at 14000×*g*. For library DNA elution by soaking, 0.4 ml of 0.3M NaCl was added to each tube, before a 4 hr rotation at room temperature. After removal of gel particles using 0.22 μm cellulose acetate filters, 10 μg of linear acrylamide (Life Technologies, USA) and 2.5 vol (approximately 1 ml) of ice-cold absolute ethanol were added. After 30 min incubation at −80°C, the eluted cDNA was precipitated by centrifugation at 4°C and maximum speed for 45 min. The pellet was washed with 1 ml of cold 80% ethanol, air dried, and resuspended in 20 μl of water. The size-selected small RNA library DNA was quantified using a Qubit apparatus with dsDNA High Sensitivity kit (Life Technologies, USA) and verified using DNA High Sensitivity 2100 Bioanalyzer chips (Agilent Technologies, USA). The library was clustered and sequenced using 76 cycles on a Genome Analyzer II (Illumina, USA) according to manufacturer's instructions.

### Data pre-processing

Details of the data pre-processing are described in Fenouil et al. (2012).

#### Quality control and filtering

In brief, all samples were sequenced on an Illumina Genome Analyzer (GAIIx for ChIP-seq and RNA-seq, or HIseq2000 for MNase-seq and H3K27ac). Quality assessment and filtering of ChIP-seq and MNase-seq sequences were performed using either the Integrated Eland software or FASTX-Toolkit (http://hannonlab.cshl.edu/fastx_toolkit/index.html) to pre-process FastQ files. Quality score and nucleotide composition at each position of the sequenced tags were assessed by box and bar plotting using FastX-Toolkit standard functions.

Quality controls (QC) and filtering of RNA samples were performed using fastQC (http://www.bioinformatics.babraham.ac.uk/projects/fastqc/), FASTX-toolkit, and Cutadapt (http://code.google.com/p/cutadapt/). Adapters were removed (Cutadapt) and a QC report was generated (fastQC). Sequences were further trimmed at nucleotide 55 and quality filtered as for DNA sample (FASTX-toolkit).

#### Processing of sequenced tags

All samples were aligned to human genome (hg19, GRCh37) using Bowtie (Langmead et al., 2009) aligner (allowing two mismatches, keeping uniquely aligned reads only). Number of tags used are shown in Table 1. For technical replicates, Eland aligned files or BAM files were merged and processed as described in Fenouil et al. (2012). Correlations between biological replicates used in this study are shown in Figure 2—figure supplement 1A. Whenever 2 replicates were not fitting a minimal good signal/noise or reproducible signal, samples were discarded from analysis and experiment reproduced. For the processing, briefly, piles of tags with same coordinates, due to artifacts of PCR or unannotated regions of the genome were removed according to a thresholding method, except for RNA-seq experiment. Uniquely aligned tags were further elongated after estimating optimal elongation size *in silico* and enabling to use the original fragment length for further processing. For ChIP-seq experiments, all samples were input subtracted and signals were scaled. For nucleosome mapping, MNAse-seq experiment in Raji was sequenced in paired-end with higher depth than ChIP-seq in Hiseq2000. Two types of analyses were applied to this data: nucleosomes density (Figure 4A, Figure 3—figure supplement 1) and nucleosomes midpoint (Figure 2D, Figure 3, Figure 3—figure supplement 1) that allow to score more specifically for depletion or positioning, respectively. For nucleosome density, paired tags were processed so to be directly connected and to retrieve original fragments (orphan tags were connected to the corresponding pairs using the estimated elongation size computed as described above). The input subtraction step was omitted. For nucleosomes midpoint analyses, the middle of elongated fragments was set as reference allowing locating the maximum signal approximately at the midpoint of the nucleosomes (dyads). For all experiments, the number of tags covering each nucleotide of the genome was computed and averaged in bins of 50 nucleotides and in bins of 10 (Figure 3—figure supplement 2C). The scores of bins were rescaled (after input subtraction when applicable) to reduce over-representation of particular genomic regions and signal/noise ratio. Finally, wig files of each corresponding biological replicates were merged.

### Bioinformatics analysis

#### Gene selection and average binding profiles

Wiggle files scores were retrieved with an in-house R script (Source code 1) for hg19 Refseq annotations and coordinates defined for selected enhancers and promoters. Overlapping annotations and those being at less than 2000 bp of another were removed. Indeed, keeping genes in vicinity of others could mix different signals leading to misinterpretation. To select Refseq genes with a significant signal, mean values distributions of Pol II isoforms and short ssRNAs at (TSS-1000 bp; TES+2000 bp) (whole gene, Figure 2C,D, Figure 2—figure supplement 5A, Figure 2—figure supplement 6A) and at TSS −/+500 bp (TSS, Figure 3, Figure 3—figure supplement 1, Figure 3—figure supplement 2) were plotted using an in-house script (Figure 2—figure supplement 1B,C, Source code 1). Gene sets of Figure 2 were selected according to each experiment mean distribution except for MNase-seq and RNA-seq whose selections were based on Pol II. For Figure 2—figure supplement 4, a less stringent threshold at 0 was used in order to study phosphorylation patterns of lower bound genes. The set of genes used in Figure 3 were also selected according to levels of Pol II at TSS (Figure 2—figure supplement 1C). Based on distribution, two Gaussian distributions were fitted using the R package mixdist (http://www.math.mcmaster.ca/peter/mix/mix.html). The threshold above which a mean value is considered as significant was set to the mean of the second Gaussian distribution. In these analyses, only genes with a length above 2 kb were kept as shorter genes tend to harbor specific profiles not reflecting the majority of coding sequences, for examples due to shorter (or lack of) introns. Moreover, histone, rRNA, snomiRNA, snoRNA, snRNA, and tRNA genes as well as outliers of short ssRNAs were removed since they also potentially modify the shapes of average profiles. Finally, a total of 1854 genes was used in Figure 3 (1846 without antisense RNA outliers).

An in-house R package retrieves signal of ChIP-seq, RNA-seq, and MNase-seq, centers them at TSS and transcription end site (TES) on a user defined selection of genes and intervals. It also retrieves all values inside each annotation, scale them to the same length, and add the half of values computed around TSS upstream and around TES downstream the annotation to obtain profiles as shown in Figure 2C. Rescaling and plotting are obtained by interpolating the retrieved values on 1000 points enabling to build a matrix on which each column will be averaged. In Figure 2—figure supplement 6A, the values were further divided into three curves representing average profiles of the top 5%, top 5–20%, and bottom 5% of genes signal.

#### Boxplots and statistical tests

The in-house package mentioned above was used to retrieve values on which means were computed. The boxplots of Figure 2E represent two sets of mean values computed on: the antisense region which was defined as covering 500 bp before TSS and the sense region as covering 500 bp after. The analysis was performed on 3201 genes defined as being the union of the set of genes retrieved for each mark. The outliers defined by the R function ‘boxplot’ by default as being all values above Q3+(1.5 × (Q3−Q1)) (interquartile range) were filtered. The remaining values were scaled between 0 and 1 before plotting. Before performing the parametric two-sided paired sample *t* test (Source code 1), the normal distribution of data was checked (data not shown). In Figure 2—figure supplement 2D, the same method was used on lowly, medium, and highly expressed genes without scaling between 0 and 1 but instead dividing by the binding values of Pol II.

Figure 2—figure supplement 2C represents mean values computed as mentioned above using only one region −/+500 bp around TSSs without removing outliers and scaling. However, outliers were not represented. The boxplots in Figure 4B, Figure 4—figure supplement 1D, and Figure 4—figure supplement 3C,D were computed similarly and divided by the corresponding mean values of Pol II. No mean values of Pol II equal to zero were detected.

#### Tyr1P peak sorting and corresponding clusters

For Tyr1P sorting (Figure 3, Figure 3—figure supplement 1), an in-house script (Source code 1) was used as described previously in Fenouil et al. (2012). Briefly, taking the genes selected as having significant level of Pol II, genes were ordered according to the relative increasing distance to the TSS of the maximal peak of Tyr1P. Other clusters show the corresponding signal of Pol II, Ser5P, Ser7P, nucleosomes, and short ssRNAs on the same genes. Heatmaps were formatted in terms of color and contrast according to sample read depth using Java TreeView software (Saldanha, 2004).

#### Definition of AT/GC content and density map

The AT content defines the presence of A or T in a particular sequence. Similarly, the GC content defines the presence of G or C in a given sequence.

The heatmaps of Figure 3—figure supplement 1A (AT and GC content) were built as follow: The corresponding fasta sequences of genes used in Figure 3 were retrieved with the R package ChIPpeakAnno (Zhu et al., 2010) in association with the Bioconductor package BSgenome.Hsapiens.UCSC.hg19 (http://www.bioconductor.org/packages/2.12/data/annotation/html/BSgenome.Hsapiens.UCSC.hg19.html). The command line RSAT tools (Turatsinze et al., 2008) enabled to retrieve the motifs W (A or T) and S (G or C) from those sequences. With an in-house script (Source code 1), the positions of motifs were converted to GFF formatted files taking into account the positions of Refseq hg19 annotations. Finally, another in-house script (Source code 1) converted those GFF to a binary matrix that was output in a format readable by Java Treeview software. This software was used as described above for color scaling.

#### Correlation of biological replicates and cross-correlation

In Figure 4D, mean values used for Spearman correlation were retrieved on selected enhancer regions (+/−2000 bp around Pol II ChIP-seq maximal signal). In Figure 2—figure supplement 1, each point represents the mean value of ChIP-seq signal of a gene on interval [TSS-1000 bp;3′+1000 bp]. For Figure 4—figure supplement 3, cross-correlation were computed −/+ 500 bp around H3K4me3 ChIP-seq maximal signal. Finally for Figure 2—figure supplement 6B, from left to right, Spearman correlations were computed on mean values at [TSS−500 bp;TSS+500 bp], [TSS+1000 bp;3'−500 bp], and [3′−500 bp;3'+1000 bp], respectively. The scripts for generating the correlations of biological replicates and tables of cross-correlation are available in Source code 1.

#### Selection of enhancers and promoters using Pol II

For the analyses shown in Figure 4 and Figure 4—figure supplement 1 (scripts and procedure to detect enhancers and promoters can be found in Source code 1), wiggle files of ChIP-seq signals of Pol II, H3K4me1, H3K4me3, and H3K36me3 were analyzed to extract enriched regions at control promoters and putative intergenic enhancers. The peak-calling was performed using an in-house script (Source code 1) fixing a threshold based on the peak height and the gap between two adjacent signals. Enriched regions separated by a distance less than a fixed max gap were merged (chosen values of thresholds and max gap are summarized in Table 1). Regions showing a combined enrichment of Pol II, H3K4me1, and H3K4me3 signal were further identified as regions of interest to build control promoters and putative intergenic enhancer sets (Figure 4—figure supplement 1A–E1P1). When a region intersected a gene annotation on the interval [TSS−2000; TSS+1000], it was defined as a ‘promoter region’ (6073 regions). To refine enhancer selection and to avoid lincRNAs promoter regions (Guttman et al., 2009; Natoli and Andrau, 2012), we removed the regions located at less than 5000 bp from any hg19 Refseq gene and harboring a significant H3K36me3 signal enrichment in vicinity that is less than 2000 bp from boundaries (747 regions, Figure 4—figure supplement 1A–E2). We further used the H3K4me3/me1 ratio to define final enhancer and promoter selections (Pekowska et al., 2011; de Almeida et al., 2011; Li et al., 2012; Natoli and Andrau, 2012). Means and relative ratios of ChIP-seq signal of H3K4me1 and H3K4me3 on intergenic enhancer candidates, on promoter candidates, and on corresponding annotated hg19 Refseq promoters (extended by ±1000 bp) were computed (Figure 4—figure supplement 1B). A threshold (green dashed line, Figure 4—figure supplement 1B) was defined to remove intergenic enhancer candidate regions with a H3K4 methylation ratio signature similar to promoters (ratio above the threshold), leading to a selection of enhancers (422 regions, Figure 4—figure supplement 1A–E3). Similarly, promoter candidates with H3K4me3 over H3K4me1 ratio below the fixed threshold were removed from the selection (5812 regions remaining, Figure 4—figure supplement 1A–P2).

In identified promoters and enhancers sets, the location of maximal signal of Pol II was defined as the centre of the region. Finally, mean values of short ssRNA signal were computed on each promoter and enhancer at centers of the regions ± 2000 bp around Pol II peak. The enhancers and promoters with outlying values were filtered from selections (390 enhancers and 4618 promoters remaining, Figure 4—figure supplement 1AE4P3). Note that using Pol II as a docking site for these analyses typically yields a rather strong nucleosome density at the middle of the promoter/enhancer area (Figure 4A). Different results can be obtained (lower nucleosome densities) when TBP is used as a docking site (de Almeida et al., 2011).

#### Selection of active enhancers and promoters using H3K27ac

Similarly to the previous section (see also Source code 1), enhancers and promoters were selected using detected peaks of H3K4me1, H3K4me3, H3K36me3, and H3K27ac (Figure 4—figure supplement 3A). This selection enables distinguishing active enhancers and promoters (having an overlapping peak of H3K27ac) from the whole selection (based on H3K4me1/3 that includes less active or poised enhancers). Although Pol II was described to be a hallmark of active and tissue specific enhancers (Koch et al., 2011), the here below described procedure enabled to retrieve a higher number of enhancers (2598; 927 active) and promoters (6057; 5946 active) giving a less stringent description of these genomic modules.

Regions harboring H3K4me1 and H3K4me3 were first split into intergenic regions without H3K36me3 (E2′, 3404) and promoter regions (P1′, 8201). Using the ratio H3K4me1/H3K4me3, 2789 enhancers (E3′) and 7147 promoters (P2′) were kept. Performing H3K27ac overlap on the above isolated regions yields 1045 active enhancers and 7030 active promoters. Regions were centered on H3K4me3 maximal values and discarding regions having short RNAs outliers finally gives active enhancers and control promoters (E5′/P5′, 927/5946) and a whole set of enhancers and control promoters (E6′/P6′, 2598/6057).

#### Tissue specificity analysis

To assess the tissue specificity of genes associated with identified enhancers and promoters of Figure 4 (Figure 4—figure supplement 1E) and Figure 4—figure supplement 3C,D, we proceeded as in Koch et al. (2011). Briefly, using bioGPS website (http://biogps.org/#goto=welcome), a Gene Atlas averaged dataset of expression values based on HGU133 array for human containing 84 tissues (Su et al., 2004) was used. The expression values of genes nearest to selected enhancers and promoters were compared to the whole dataset and the significance of differences was assessed with a nonparametric statistical Mann-Whitney-Wilcoxon test. Bars of expression levels were sorted by p-values and the 10 most differentially expressed tissues are shown. Scripts generating the tissue specificity barplots are available in Source code 1.

#### Gene expression analysis

Microarrays data of expression in Human Raji cell line was retrieved on Array Express database (E-GEOD-46873). Corresponding symbols between array chip and human hg19 Refseq annotations were downloaded from Ensembl Biomart (Release 73, Flicek et al., 2013).

Replicates were merged by taking the mean of expression. When several probes indicated expression of a single Refseq gene, the median of expression was attributed to the corresponding Refseq ID. Refseq genes were then ordered by expression removing genes at less than 2 kb from another and particular categories of genes were removed as described in ‘Materials and methods–Gene selection and average binding profiles’. Corresponding ChIP-seq and short ssRNA-seq signals were retrieved on the ordered genes and represented with heatmaps at 1 kb around TSS (Figure 2—figure supplement 2). For profiles shown in Figure 2—figure supplement 2B, outliers were discarded. As mentioned above, colors were scaled using Java Treeview software. Profiles (Figure 2—figure supplement 2B) and boxplots (Figure 2—figure supplement 2CD) were built as described in ‘Materials and methods–Gene selection and average binding profiles and Boxplots and statistical tests’. Input files and scripts used can be found in Source code 1.

#### CTD isoforms and nucleosomes midpoint maximal peaks spatial organization analysis

Binary matrices of 2000 interpolated ChIP-seq values shown in Figure 3A and Figure 3—figure supplement 1A in Java Treeview format were used as input. They were reduced to values at ± 500 bp around TSS and the list of genes limited to group 1. Maximal value indexes of Tyr1P signal were first retrieved. To avoid ambiguities in interpretation in our relative Pol II and isoforms positions in class I (Figure 3—figure supplement 2), we analyzed those genes that belonged to class I and that clearly featured AS transcription. For this, mean values of short ssRNA experiments were computed at −50/+100 bp around Tyr1P detected maximal peaks. Genes having a significant level of antisense RNAs were kept using the mean distribution and thresholding as described in ‘Materials and methods–Gene selection and average binding profiles’. Only annotations with a level of antisense RNAs higher than sense RNAs were kept performing a one sided nonparametric Mann-Whitney-Wilcoxon statistical test (p-value <0.05). A total of 529 genes were selected. Then, maximal peaks detection for the other marks was computed at −100/+100 bp around Tyr1P signal. For each maximal peak of each experiment shown in Figure 3—figure supplement 2, the distances to maximal peaks of Tyr1P and Pol II were computed. The R package rgl (http://rgl.neoscientists.org/about.shtml) was used for 3D representation of the maximal peaks according to the distance to TSS, to Pol II and their values.

To assess if distances retrieved were not originating from background noise (Figure 3—figure supplement 2C), distances to Pol II of selected genes of (A) on 500 bp before TSS (representing the AS region) were compared to the total set of genes (keeping only genes at more than 2000 bp from any RefSeq annotations). Kernel density estimates were computed using R. A lower resolution of 10 bp was used (compared to 50 bp of Figure 3—figure supplement 2A,B). Scripts and procedures to generate figures of the spatial analysis can be found in Source code 1.

## Additional files

Source code 1. Folder containing the different scripts used in this study. **DOI:** http://dx.doi.org/10.7554/eLife.02105.022

### Major datasets

The following datasets were generated:

DescostesN, HeidemannM, SpinelliL, SchüllerR, MaqboolMA, FenouilR, KochF, InnocentiC, GutM, GutI, EickD, AndrauJ, 2014, Pol II, http://www.ncbi.nlm.nih.gov/geo/query/acc.cgi?acc=GSE52914, Publicly available at NCBI Gene Expression Omnibus.

DescostesN, HeidemannM, SpinelliL, SchüllerR, MaqboolMA, FenouilR, KochF, InnocentiC, GutM, GutI, EickD, AndrauJ, 2014, H3K4me1, http://www.ncbi.nlm.nih.gov/geo/query/acc.cgi?acc=GSE52914, Publicly available at NCBI Gene Expression Omnibus.

DescostesN, HeidemannM, SpinelliL, SchüllerR, MaqboolMA, FenouilR, KochF, InnocentiC, GutM, GutI, EickD, AndrauJ, 2014, H3K4me3, http://www.ncbi.nlm.nih.gov/geo/query/acc.cgi?acc=GSE52914, Publicly available at NCBI Gene Expression Omnibus.

DescostesN, HeidemannM, SpinelliL, SchüllerR, MaqboolMA, FenouilR, KochF, InnocentiC, GutM, GutI, EickD, AndrauJ, 2014, H3K36me3, http://www.ncbi.nlm.nih.gov/geo/query/acc.cgi?acc=GSE52914, Publicly available at NCBI Gene Expression Omnibus.

DescostesN, HeidemannM, SpinelliL, SchüllerR, MaqboolMA, FenouilR, KochF, InnocentiC, GutM, GutI, EickD, AndrauJ, 2014, H3K27ac, http://www.ncbi.nlm.nih.gov/geo/query/acc.cgi?acc=GSE52914, Publicly available at NCBI Gene Expression Omnibus.

DescostesN, HeidemannM, SpinelliL, SchüllerR, MaqboolMA, FenouilR, KochF, InnocentiC, GutM, GutI, EickD, AndrauJ, 2014, Tyr1P, http://www.ncbi.nlm.nih.gov/geo/query/acc.cgi?acc=GSE52914, Publicly available at NCBI Gene Expression Omnibus.

DescostesN, HeidemannM, SpinelliL, SchüllerR, MaqboolMA, FenouilR, KochF, InnocentiC, GutM, GutI, EickD, AndrauJ, 2014, Ser2P, http://www.ncbi.nlm.nih.gov/geo/query/acc.cgi?acc=GSE52914, Publicly available at NCBI Gene Expression Omnibus.

DescostesN, HeidemannM, SpinelliL, SchüllerR, MaqboolMA, FenouilR, KochF, InnocentiC, GutM, GutI, EickD, AndrauJ, 2014, Ser5P, http://www.ncbi.nlm.nih.gov/geo/query/acc.cgi?acc=GSE52914, Publicly available at NCBI Gene Expression Omnibus.

DescostesN, HeidemannM, SpinelliL, SchüllerR, MaqboolMA, FenouilR, KochF, InnocentiC, GutM, GutI, EickD, AndrauJ, 2014, Ser7P, http://www.ncbi.nlm.nih.gov/geo/query/acc.cgi?acc=GSE52914, Publicly available at NCBI Gene Expression Omnibus.

DescostesN, HeidemannM, SpinelliL, SchüllerR, MaqboolMA, FenouilR, KochF, InnocentiC, GutM, GutI, EickD, AndrauJ, 2014, short RNAs, http://www.ncbi.nlm.nih.gov/geo/query/acc.cgi?acc=GSE52914, Publicly available at NCBI Gene Expression Omnibus.

DescostesN, HeidemannM, SpinelliL, SchüllerR, MaqboolMA, FenouilR, KochF, InnocentiC, GutM, GutI, EickD, AndrauJ, 2014, MNase, http://www.ncbi.nlm.nih.gov/geo/query/acc.cgi?acc=GSE52914, Publicly available at NCBI Gene Expression Omnibus.

DescostesN, HeidemannM, SpinelliL, SchüllerR, MaqboolMA, FenouilR, KochF, InnocentiC, GutM, GutI, EickD, AndrauJ, 2014, input, http://www.ncbi.nlm.nih.gov/geo/query/acc.cgi?acc=GSE52914, Publicly available at NCBI Gene Expression Omnibus.
